# The untranslatability of environmental affective scales: insights from indigenous soundscape perceptions in China

**DOI:** 10.1038/s42949-025-00228-6

**Published:** 2025-06-14

**Authors:** Duotuo Wu, Rumei Han, Ruining Zhang, Xinhao Yang, Yuan Zhang, Jian Kang

**Affiliations:** 1 Liaoning Provincial Key Laboratory of Eco-Building Physics Technology and Evaluation, Shenyang, China; 2 School of Architecture and Urban Planning, Shenyang Jianzhu University, Shenyang, China; 3 School of Science, Shenyang Jianzhu University, Shenyang, China; 4 School of Architecture, Tianjin University, Tianjin, China; 5 UCL Institute for Environmental Design and Engineering, The Bartlett, University College London, London, UK

**Keywords:** Psychology, Environmental social sciences, Environmental studies, Geography, Language and linguistics

## Abstract

Soundscapes significantly impact human well-being, yet standard Western-based Soundscape Affective Quality (SAQ) scales may not accurately represent culturally specific perceptions. This study explored structural differences in soundscape emotional experiences between Chinese and Western cultures using an indigenous approach. An Indigenous Chinese SAQ (ICSAQ) scale was developed through perceptual evaluations of 132 representative Chinese soundscape excerpts by 264 participants from 30 provinces, employing 108 culturally relevant descriptors. Principal component analysis revealed two dimensions—Comfort and Richness—differing notably from the global Pleasantness-Eventfulness model. Comparison with the translated global SAQ scale showed significant measurement bias, with the imported scale overestimating positive emotions and activation levels. Regression analyses confirmed the ICSAQ scale’s stronger interpretability regarding objective acoustic indicators. The findings highlight critical limitations of translation-based environmental affective measurements, emphasizing the necessity of culturally appropriate assessment tools for inclusive urban environmental management.

## Introduction

Emotion is a complex psychological and physiological state that drives subjective experiences, physiological responses, and expressive behaviours^1^. According to Constructionist theories of emotion, emotion categories are non-entitative, with no consistent mapping between specific emotion categories and dedicated biological mechanisms (constructed by more general brain networks)^2^. As constructed phenomena, emotions involve allostasis, abstract categorisation, and social learning^3–5^. Humans typically use symbolic language to support the recognition, understanding, and expression of emotions^6^, integrating complex physiological and psychological responses into specific emotion categories. Language-supported category access can lead to different experiences derived from a multidimensional feature space (emotional states, physiological responses, autonomic nervous system activity, and facial behaviours)^7–9^. Jackson et al. used a colexification approach (i.e., the phenomenon in language where the same word is used to name semantically related concepts) to reveal extensive differences in emotional semantics across 20 global language families—different patterns of association exist in the networks of emotion concept colexification across language families^10^.

Russell’s circumplex model of affect provides a systematic representation of the processes of perception, cognition, and emotional response to external information. It emphasises that affect is essentially a neurophysiological state composed of two dimensions: valence (pleasure vs. displeasure) and arousal (physiological activation)^11^. These two dimensions combine to form core affect^12^, which serves as the foundation for emotional responses and represents a fundamental and intrinsic emotional experience. All emotional states are mapped as points in a two-dimensional space defined by valence and arousal, thus being interpreted as specific emotional concepts. Since valence and arousal arise from independent neurophysiological systems, based on the capacity for biological evolution, various cultures may universally distinguish emotional states based on these dimensions^10^.

**Fig. 1:**
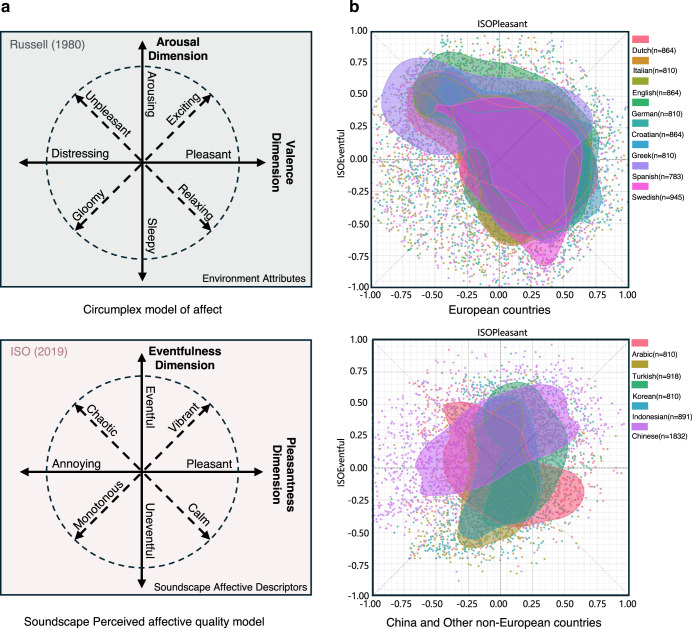
Background framework. **a** Frameworks of affective quality in different domains: The green framework represents the environmental affective quality framework and its constructed model, also known as the structure of core affect^12^. The red framework represents the soundscape affective quality framework and its constructed^18^; **b** Soundscape Affective Quality (SAQ) Assessment results of 13 languages that showed “high confidence” results. Each point represents a sample, and the shaded areas represent contours that enclose 50% samples. It can be seen that European countries with close geographical proximities showed similar distributions. (Data Source: Soundscape Attributes Translation Project (SATP) Dataset^26^).

Sound is a powerful emotional elicitor. In urban spaces, soundscapes are composed of multiple dynamic sound sources that exhibit spatiotemporal variability^13–15^. The perception of soundscapes is a continuous and dynamically evolving process, involving both conscious and unconscious evaluations^16^. Through this process, individuals interpret the acoustic environment within a given context, encompassing physiological, psychological, social, and cultural dimensions. Even a single sound source can elicit multidimensional emotional experiences due to the complexity of perception. To measure the intertwined emotional cues of soundscapes, researchers in soundscape studies have used the circumplex model of affect attributed to environments as a starting point^17^, providing an effective tool—the Soundscape Affective Quality (SAQ) model—to identify and quantify emotional responses elicited by specific soundscapes. This model defines eight Soundscape Affective Descriptors (SADs) within a two-dimensional circular space of Pleasantness–Eventfulness^18–20^. Similarly, the sense of Pleasantness reflects the positive or negative affect elicited by the soundscape, while the sense of Eventfulness reflects the alertness or energy state induced by the soundscape. Figure 1a illustrates a comparison between the circumplex model of affect and the SAQ model. However, compared to the general circumplex model of affect, the emotion measurement instrument developed for specific domains (such as this one for urban soundscape studies) can more accurately capture the complex emotional experiences associated with particular stimuli. This approach has also been confirmed in the music and olfactory fields^21,22^.

Culture shapes individuals’ emotional experiences and expressions in different contexts through unique frameworks for understanding emotions and norms for expressing them^23^. Similarly, cultural differences affect the way individuals perceive, express, and evaluate the SAQ. In this context, careful consideration needs to be given to the “transplantation” of specific measurement instruments or theoretical frameworks from one cultural context to another, i.e., “imposed-etic approach”. In a recent global collaboration, the Soundscape Attributes Translation Project (SATP)^24,25^, a list of standardised soundscape affective descriptors was translated into 18 languages. Native speakers from various countries evaluated the same soundscape stimuli using their respective language versions of the SAQ scale. However, the results revealed significant differences in the affective quality ratings of soundscapes between Chinese participants and those from other countries, as illustrated by the density plot of soundscape quality ratings^26^ (Fig. 1b). This observation prompted an in-depth consideration of possible reasons: first, Chinese soundscape emotion may have significant structural differences from other cultures, which may stem from differences in soundscape affective semantic space; second, differences in affective semantic space may lead to the possibility that the imported instrument models may cause bias in the assessment of SAQ.

From an emic perspective, it is essential to explore the emotional salience and motivational forces within cultural scripts rather than assuming these saliences and forces. This involves adopting an insider’s view of culture, presenting human behaviour and subjective experiences from the perspectives of actors, intenders, and subjects of attention, and exploring “what life is like there—what features are salient to its inhabitants”^27,28^.

Herein, through the emic approach, we explore the Chinese soundscape affective semantic space from specific cultural phenomena and experiences based on local input items (Chinese SADs) and representative soundscape excerpts (SEs) of Chinese urban open spaces and validate their robustness through further grouping into sub-dataset. The structure of Chinese SAQ is established based on Chinese soundscape affective semantic space, and the assessment differences between the indigenously derived (indigenous Chinese SAQ scale, ICSAQ scale) and the imported SAQ scales (translated global SAQ scale, TGSAQ scale) are compared. The results showed the significant structural differences in soundscape emotional experiences between Chinese and Western cultures, confirming the necessity of developing culturally appropriate environmental affective assessment tools.

## Results

### Construction of semantic space of soundscape affective quality in the Chinese context

**Fig. 2:**
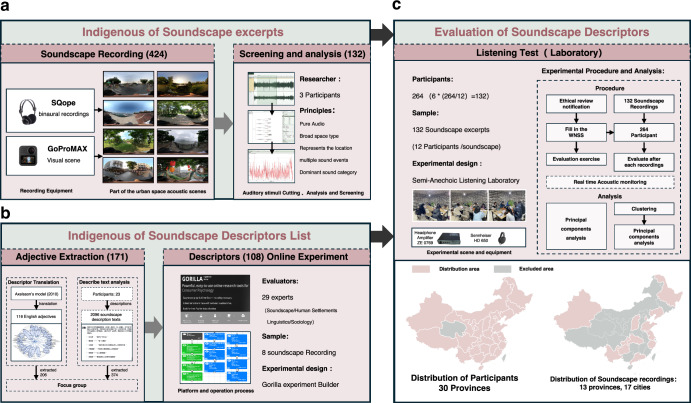
Process flow of the research method. **a** Collection and analysis of indigenous soundscape excerpts (SEs); **b** Indigenous soundscape descriptors solicitation and screening; **c** Listening experiment.

To carry out the experiment from an indigenous perspective, SEs and a list of SADs had to be established locally. The methodological framework is shown in Fig. 2. To capture the diverse and characteristic soundscape of China, we recorded 424 SEs from 13 provinces of China that each comprises of a 30-second binaural audio and a panoramic shot of the surrounding visual context. These SEs were screened, and 132 high-quality SEs that represented 17 urban forms were selected for subsequent experiments. The indigenous SAD list was then created through a process of adjective extraction, focus group interviews, and online screening experiments. This created a list of 108 potential SADs that are suitable for describing Chinese soundscapes (refer to Table S9 for the explanations of all descriptors).

With the indigenous SEs and SADs in hand, a listening experiment was carried out. The participants rated the SE-SAD pairs in terms of how well the SAD matched their perception of the soundscape. The SADs assessed included the 108 developed here and eight translated in the SATP project.

**Fig. 3:**
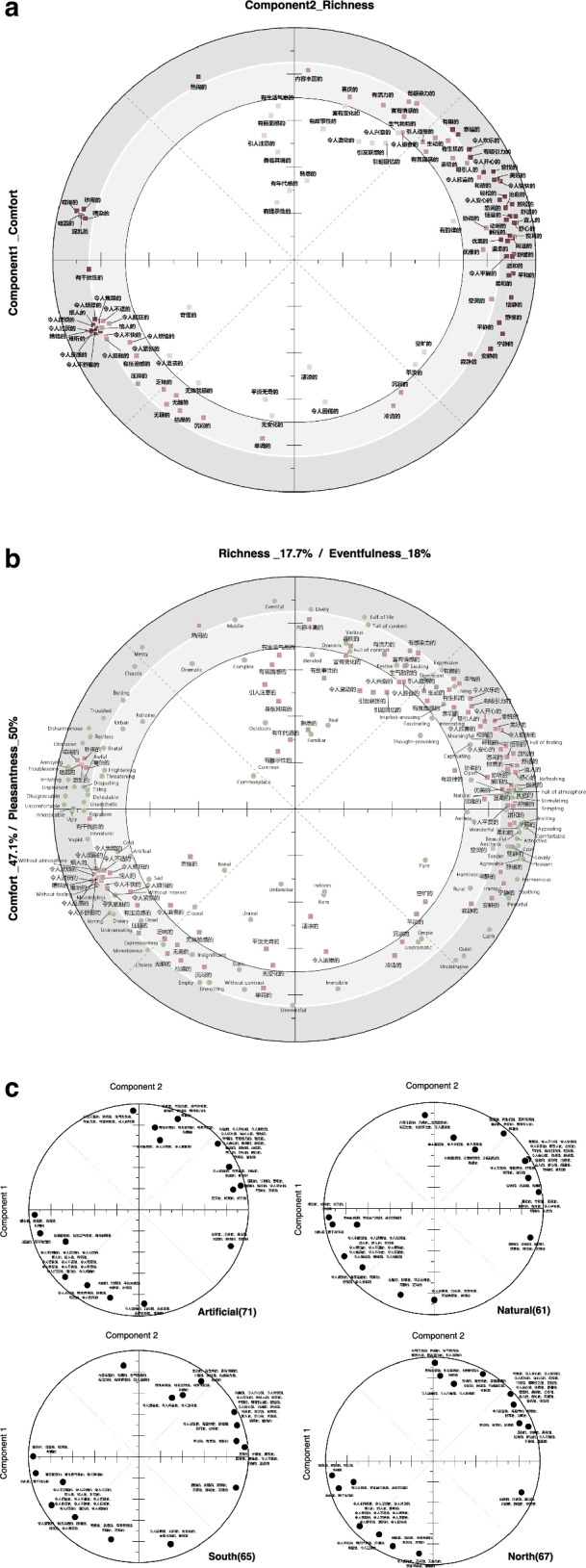
Soundscape semantic space models. **a** Soundscape semantic space of China: Loadings of the 108 Chinese soundscape affective descriptors (SADs) in components 1 and 2. The figure is divided into three zones according to the length of the component loading vectors of the 108 Chinese SADs (*Va*: the distance to the origin), Zone 1 (light red square); Zone 2 (red square); and Zone 2 (dark red square); where *Va2* represents the variance of each attribute that the corresponding components; **b** The soundscape semantic spaces of Europe^19^ and China depict the loadings of 116 English soundscape affective descriptors (SADs) and 108 Chinese SADs across components 1 and 2. Green circles represent the English SADs, while red squares denote the Chinese SADs; **c** Soundscape semantic space of the sub-datasets: Displays the loadings of the 16 clustering descriptors in components 1 and 2.

For the dataset acquired, principal component analysis (PCA) was then used to analyse the outcome of the listening experiment. The Kaiser‒Meyer‒Olkin value was 0.847 ( > 0.6). The results of Bartlett’s test of sphericity were significant (*χ*^2^ = 21695.1, *p* < 0.001), suggesting that the dataset was suitable for PCA. The affective state through two-demission circumplex structure description and classification has been widely empirically supported^12,29^, and considering the special needs and practical goals of soundscape research (based on the ISO-12913-3 two-demission circumplex model of SAQ) and exploring soundscape emotion in the Chinese context, we chose to extract two principal components (PCs) as the basis of our analysis. The results show that PC 1 and PC 2 explain 47.1% and 17.7% of the variance in the dataset, respectively, and these two PCs together explain 64.8% of the total variance, which can effectively represent the structure and characteristics of the original dataset. Figure 3a shows the component loadings of the two PCs. The SADs on each PC can be divided into three zones according to the size of the loadings: Zone 1: *Va* < 0.70; Zone 2: 0.70 < *Va* < 0.85; Zone 3: *Va* > 0.85, where *Va* denotes the variance of each SAD that can be interpreted by the corresponding PC. The SAD in Zone 1 does not explain the PC well.

The two PCs each embody a different dimension of the soundscape emotional experience. This is based on the similarity of the revealed results to the emotion construct^12,30^. In the circular space, the SADs yirende/宜人的, shushide/舒适的, shuxinde/舒心的, xuannaode/喧闹的 and hunluande/混乱的 (in descending order of their component loadings on the PC) had the highest interpreted rates on PC 1. When soundscapes are described as yirende/宜人的 or shushide/舒适的, this may indicate that the soundscape has a positive effect on the individual. Conversely, if the soundscape is described as hunluande/混乱的 or other SADs, this may indicate that the soundscape is causing the individual to experience negative affect such as stress and anxiety. Together, these SADs describe the positive or negative affective sate elicited by the soundscape; therefore, PC 1 was labelled 舒适感 (Comfort). The highest SADs in PC 2 include fengfude/丰富的, renaode/热闹的, dandiaode/单调的, wubianhuade/无变化的 and chenmende/沉闷的. fengfude/丰富的 and renaode/热闹的 indicate the variety and activity of the soundscape, which tends to stimulate physiological responses and mental alertness. dandiaode/单调的, wubianhuade/无变化的 and chenmende/沉闷的, on the other hand, may result from low levels of physiological and psychological responses. Together, these adjectives describe the differences in the soundscape in terms of triggering physiological activity and mental alertness; hence, PC 2 is labelled 丰富感 (Richness).

We name this model, as presented in Fig. 3a, the semantic space of SAQ of China. The simple plot of the affective states within the Cartesian space formed by the underlying dimensions suggests that this is a circular structure rather than a simple linear structure. In this circular structure space, Chinese SADs are meaningfully arranged, attributing various affective states of soundscapes to different combinations of Comfort or Richness. The first quadrant contains soundscapes that are generally perceived as comfortable and rich, while the opposite quadrant contains various annoying soundscapes.

### Soundscape affective quality comparisons between regions and within China

The current globally adopted soundscape perception scale originated from the soundscape semantic space model developed by Axelsson et al. In Europe^19^. Among the two PCs in that model, PC 1 (Pleasantness) explained 50% of the variance, which was best explained by “uncomfortable”, “comfortable”, “appealing”, “disagreeable” and “inviting”. PC 2 (Eventfulness) explains 18% of the variance, which is best explained by “eventful”, “lively”, “uneventful”, “full of life” and “mobile”.

The SAD alignment patterns in the circumplex space reveal differences in the composition of the soundscape affective state across cultures. Specifically, there are fewer SADs in the second and fourth quadrants of the Chinese soundscape semantic space. In addition, SADs such as hunluande/混乱的, xuannaode/喧闹的, chaonaode/吵闹的, shushide/舒适的, pinghede/平和的, wenhede/温和的 and xianshide/闲适的 are close to the axes, which is not the case in the European space, as shown in Fig. 3b. Caution needs to be exercised in interpreting the specifics of PCs. According to the soundscape semantic space, potential dimensions are given different labels, but Comfort and Pleasantness both reflect the positive or negative tendency (valence) of soundscape emotional experience, whereas Richness and Eventfulness both represent the intensity of soundscape affective activation (arousal).

Based on the regional distribution and urban spatial types, we grouped the data into two sub-datasets in two ways: South and North, and natural and artificial. Each pair would add up to the complete dataset. Cluster analysis of all samples resulted in the identification of 16 SAD clusters. In the four datasets, PAC was performed on these 16 clusters respectively. In the PAC of each dataset, two PCs were obtained, and all datasets passed the KMO and Bartlett tests. The results show that the two PCs of each of these four sub-datasets can all be labelled as Comfort and Richness. The “variance explained” parameter for the two PCs ranges between 40.9–50.3% and 26.1–39.8%, respectively, and together explain 76.3–80.7% of the total variance. In addition, the SADs under the four types of contexts show very similar distributions in the soundscape semantic space, as shown in Fig. 3c.

### Construction of an indigenous Chinese soundscape affective quality scale

**Fig. 4:**
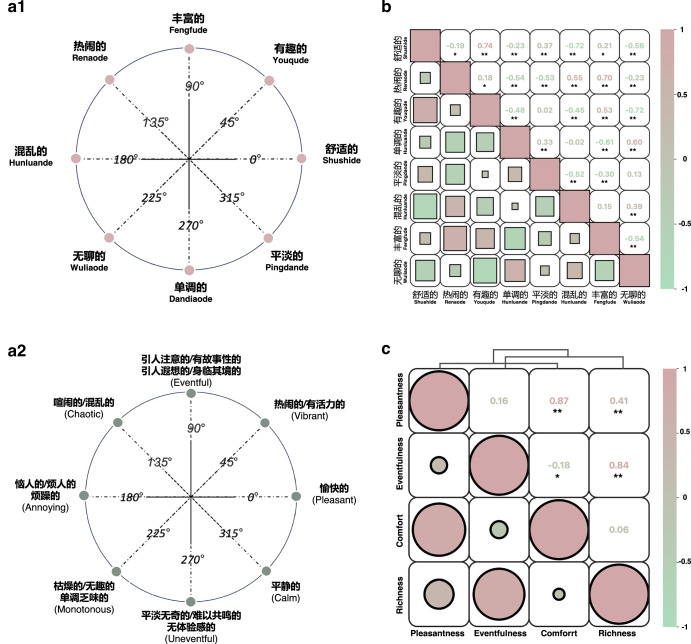
Structure of soundscape affective quality scales and correlation heatmap. **a1** Indigenous Chinese soundscape Affective quality scale (ICSAQ scale). Displays the eight descriptors and their circular arrangement; **a2** Translated global SAQ scale (TGSAQ scale)^25^. Displays the eight descriptors and their circular arrangement; **b** Analysis of the indigenous SAQ structure. Presents a heatmap of the correlation between the SAQ and clustering among the eight SADs; **c** Correlation analysis of indigenous and imported scales. Shows the heatmaps of the correlation between the two PCs and the results of cluster analysis.

Figure 4a summarises the proposed Chinese SAQ structure, in which eight SADs are placed in a circular order at approximately 45° intervals, forming an equally spaced circular structure. This structure provides a network of testable propositions to which all SADs are related, the effective quality attributed to a particular soundscape can fall at any point in space, and any SAD can be represented as a vector originating from the center of the circle. The model does not aim to capture all emotion but rather aims to provide a means of describing or conceptualising people’s perceptions of SAQ at the most general level. There has been empirical evidence on this class of models, for example, that their affective space is bipolar, and the two principal axes are independent and of comparable significance in the semantic space^31,32^. The SAQ structure discussed is limited to eight SADs, but theoretically, the circumplex model allows for infinite subdivision, and any of the SADs of the soundscape semantic space can be used for measurement. The order in which these SADs form the circumplex is shown in Fig. 4a: pingdande/平淡的, youqude/有趣的, wuliaode/无聊的, renaode/热闹的, shushide/舒适的, hunluande/混乱的, fengfude/丰富的and dandiaode/单调的. The ring structure can also be defined in independent dimensions: “shushide/舒适的-hunluande/混乱的” and “fengfude/丰富的-dandiaode/单调的”.

If the eight SADs shown are exactly 45° apart and measured without error, their correlation should conform to a specific pattern in the correlation matrix^33^. However, as this is psychology and not geometry, in practice, owing to the complexity of mental structures, it does not necessarily follow a strict circumplex structure^34,35^. As shown in Fig. 4b, the actual correlation matrix obtained is very close to the expected value of the circular ordering of the variables. This process produced four bipolar scales whose Cronbach’s alpha estimates were very adequate (Supplementary Information Table S1), and the correlations between the scales were roughly in line with the expected pattern of correlation. The reliability of the pairs ranged from 0.70 to 0.84 ( > 0.70 is considered high).

### Measurement bias of the translated global SAQ scale

When SAQ measurement instruments are applied to another culture, issues of measurement bias and equivalence become important^36,37^. Here we compared the developed ICSAQ scale with the translated scale (TGSAQ scale). The affective quality scores of the 132 SEs were measured using the two scales (Fig. 4a). Pearson correlation (Fig. 4c) revealed high correlation coefficients between the Comfort and Pleasantness dimensions (0.887) and between the Richness and Eventfulness dimensions (0.843). This suggests that the two instruments are effective in measuring the same construct. However, this does not mean that the two scales are equivalent. The underlying dimensions of the two scales are assigned different labels, and structural bias may occur when the constructs to be measured do not overlap exactly across cultures^38^. In addition, there are differences in some particular SADs, and different emotional response styles may give rise to instrument bias (method bias) and item bias. Any combination of construct, method, or item bias that may be present in TGSAQ scale can give rise to bias in test interpretation^39^. These potential biases are assessed below.

**Fig. 5:**
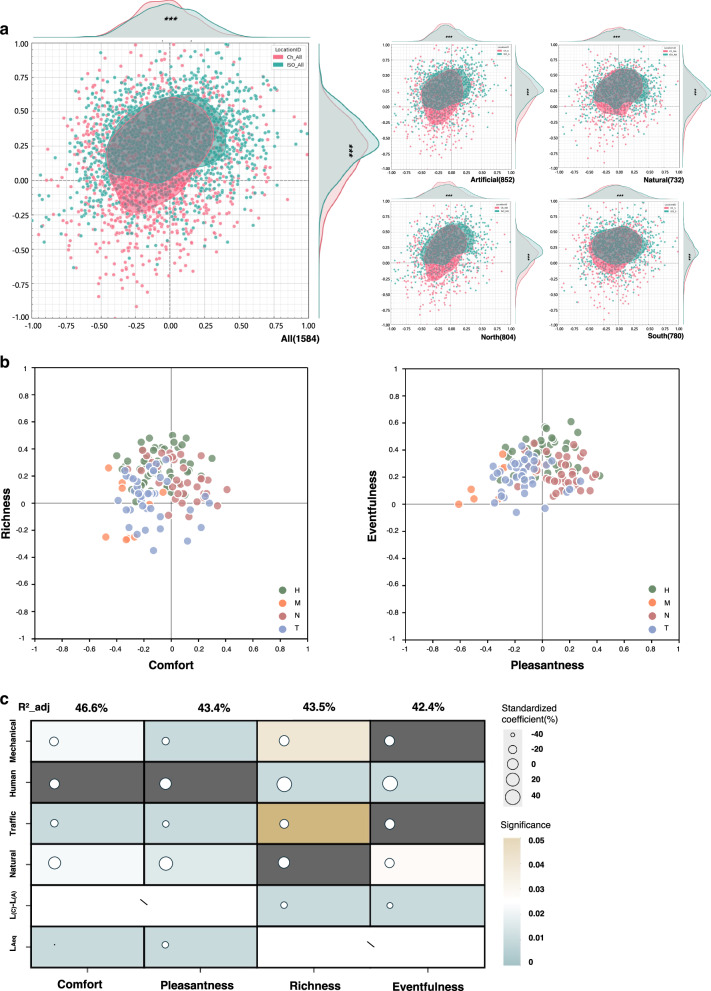
Comparison of evaluation results between the indigenous and imported SAQ scales. **a** Density plots of SAQ scores of the complete dataset (*N* = 1584) and the four sub-datasets. (*) denotes significance levels from the *t* test. The red plot represents the evaluation results of soundscape affective quality measured using the ICSAQ scale, while the green plot shows the results measured using the TGSAQ scale; **b** Component scores for 132 soundscape excerpts (SEs) categorised by dominant sound types. Shown are the component scores for Comfort and Richness, as well as Pleasantness and Eventfulness. Colours represent dominant sound categories. **c** Multiple linear regression analysis. Four models are examined: Comfort, Pleasantness, Richness, and Eventfulness model. Six independent variables are trialled for inclusion: dominance by four types of sounds (each is a dichotomous variable), *L*_(C)_ *- L*_(A)_, and *L*_Aeq_. Variables included in the regression model have their standardised coefficients and significance represented. The cell colour denotes the significance, *p ≤ 0.05, **p ≤ 0.01, ***p ≤ 0.001. Size of the circles indicates the standardised coefficients.

The Shapiro‒Wilk test was performed on the whole dataset, and the results revealed that all the variables conformed to the characteristics of a normal distribution (*p* > 0.05). On this basis, *t* test_rel (i.e., paired samples *t* test) was used to compare the results of the two measurement instruments on the same samples, and significant differences (*p* < 0.05) were found. Density plots of the SAQ scores were plotted using Soundscapy^40^, as shown in Fig. 5a, to explore possible cases of interpretation bias.

The ICSAQ scale results revealed a broader and more homogeneous near-circular distribution of the SAQ, covering all quadrants. Specifically, the 95% confidence intervals (CIs) were −0.36 to 0.25, with a median of −0.11 for Comfort, and −0.24 to 0.42, with a median of 0.17 for Richness. In contrast, the TGSAQ scale results indicated a predominantly elliptical distribution of the SAQ along 45 degrees. The 95% CI of Pleasantness was −0.34 to 0.29, with a median of −0.04, and Eventfulness had a 95% CI of 0.05 to 0.48, with a median of 0.27. This distribution was similar to the findings of Chinese density plots of soundscape quality in SATP^25^.

A comparison of the distributions of the results of the two measurement instruments across all datasets revealed that the ICSAQ scale, which originated from indigenous development, was able to capture more variation or more detailed soundscape features. In contrast, TGSAQ scale failed to capture soundscapes effectively about pleasant, calm and eventful. Moreover, TGSAQ scale overestimates the positive affect of the SAQ and the intensity of SAQ activation, which may mask potentially negative or complex affective components of the soundscape. In addition, TGSAQ scale ratings show a clear concentration on the affect activation dimension of soundscapes, with narrow score ranges that fail to effectively capture a large amount of soundscape detail.

The results of the assessment of the affective quality of the four dominant sound source types show a clear pattern (Fig. 5b). Unsurprisingly, the acoustic environment dominated by traffic sounds and mechanical sounds was generally considered chaotic. Evaluations of natural sounds and human sounds, on the other hand, showed a diverse distribution of affect. Natural sound-dominated acoustic environments are usually perceived as more acoustic comfort, but partially natural sound-dominated SEs are uncomfortable. In Chinese urban spaces, the types of sound sources are complex and intertwined, and even scenes dominated by natural sounds may contain other types of sound sources, which may explain why they are sometimes perceived as less comfortable. SEs dominated by human sounds are often perceived as richer. However, the relationship between the sound source type and the soundscape affective dimension is not absolute. This comparison showed that the ICSAQ scale had a greater degree of differentiation in the Chinese context.

By building a multiple regression model, we compared the influence of the acoustic indicators on each dimension of the SAQ, as shown in Fig. 5c. The results show that the Comfort model has an explanatory variance of 46.6%, which is higher than the Pleasantness model’s 43.4%. Similarly, in the Richness and Eventfulness models, the Comfort model has an explanatory variance of 43.5%, which is higher than the Eventfulness model’s 42.4%.

## Discussion

Emotion is conceptualised through social learning, a process influenced by cultural evolution. Certain emotions have been shown to differ culturally^41–43^. In the soundscape semantic space, the cultural differences in soundscape emotional concepts are clearly reflected, with Comfort as the principal axis of Chinese soundscape quality (as opposed to Pleasantness), reflecting that Chinese people tend to consider affective states such as “comfort” and “calmness” as more positive in the emotional experience of the soundscape. This emotional preference can be traced back to the traditional values of tranquillity and harmony in Chinese culture and has been validated in psychological studies, where individuals from Western cultures are more inclined toward high arousal positive affect (feeling excited and enthusiastic), whereas individuals from Eastern cultures typically exhibit lower levels of affect arousal (feeling of relaxation and peace)^44,45^. Moreover, cultural differences in the ideal affect emerge early in life, as shown by European–American children preferring more arousal states than children in Taiwan in best-selling storybooks^46^.

Although there are significant differences in the acoustic environment in different regions and types of urban space in China, these differences do not fundamentally change the structure of the semantic space of the soundscape, which reflects the way individuals perceive, express, and evaluate the SAQ mostly depends on the national culture. Culture is defined as “shared elements that provide the standards for perceiving, believing, evaluating, communicating, and acting among those who share a language, a historic period, and a geographic location”^47^.

Culture defines and produces emotional words^48^, guiding individuals through language to communicate around and share the meaning of shared perceptual categories, enabling social groups to apply uniform “rules” to recognise aspects of the environment^49^. The significant influence of language on the acquisition of emotional concepts and perceptual categories has been widely demonstrated^50–52^. Even when individuals in different cultures are faced with the same emotional stimuli, the form and internal structure of their emotional response can vary^53^. Therefore, soundscape experiences across cultures can be more accurately assessed by considering the cultural context, prompting deeper consideration of methodological distinctions such as emic versus etic approaches in cross-cultural research.

Such cultural considerations raise important methodological issues for cross-cultural psychology, where debate between etic (culturally universal) and emic (culturally specific) methodologies can be traced back to Berry’s reflections on comparative research paradigms^54^. The etic approach advocates for the use of analytical frameworks that transcend specific cultures, employing standardised tools to identify universal laws of human psychology and behaviour^55^, and ensuring the universality of measurement tools through tests of structural equivalence^37^. In contrast, the emic approach emphasises the construction of theories from within cultures, capturing the unique experiences of specific groups through localised concepts^56,57^.

The imposed-etic strategy adopted by the SATP project^24^ assumes that the underlying theories and conceptualisations are universally applicable and can “maximise the chances of discovering cross-cultural comparisons, excluding culture-specific dimensions”^58^. However, this strategy may lead to bias on the basis of a selective subset of universal structures. The TGSAQ scale was found to provide somewhat valid assessments in this study, but it was weak in its ability to portray certain specific soundscapes and resulted in biased assessments of positive affect and degree of activation. Despite the fact that the TGSAQ scale has been validated by the bilingual test-retest method^25^ and that differential item functioning is able to reduce interpretation bias and obtain good test items to some extent^59^, without sufficient evidence of configural equivalence, metric equivalence, and scalar equivalence, cross-cultural mean comparisons may generate misleading results, which leads to false cultural conclusions and inaccurate cultural stereotypes^60^. However, interestingly, the items that exhibit differential item functioning across cultures and those that deviate from scalar equivalence may precisely explain the existence of cultural differences^38^.

As Kim stated, the goal of indigenous psychology is “to identify knowledge as understood and experienced by people within a culture”^61^. In the past, owing to the lack of localised instruments originating from within a culture, SAQ assessments have relied on existing external global instruments. Although these scales have some cross-cultural validity, the combination of the relative inherent structures in particular cultures can enable the capturing of specific soundscape emotional concepts more fully, given the cultural differences. Variations in emotional concepts are associated with the geographic proximity of language families^10,62,63^. Considering the geographic variability of cultures using the emic approach to construct the SAQ structure from the bottom up allows for a better understanding and description of the culture-specific soundscape emotional experience from an intra-cultural perspective.

The emotional labelling effect suggests that individuals can better understand complex affective states when they break them down into recognizable labels through natural language and can accurately identify and name emotions^64,65^. Thus, by utilising the “material” composition assessment instrument on the ontology map of the Chinese soundscape’s affective semantic space, the SAQ can be assessed more sensitively and with better discrimination between types of sound sources. The emic approach provides a perspective that reveals cultural differences in soundscape emotional concepts and similarities in soundscape emotional experiences between imported Western instruments and indigenous instruments in Chinese culture. This is further evidence that the Chinese are “constructing the socio-perceptual world differently”^66,67^. However, as Yang and Bond imply, imported and indigenous instruments may be useful for different theorisations in the same culture while at the same time being useful^68^. As a pioneering effort in non-Western contexts, the ICSAQ scale not only broadens cross-cultural SAQ research beyond mere translations but also prompts a deeper inquiry into whether such untranslatability extends beyond soundscapes to other environmental affective domains. This methodological shift towards indigenous frameworks reveals broader challenges in translating emotional constructs, suggesting that similar issues of cultural specificity may impact the assessment of environmental affective quality more generally.

Reflecting on the challenges of translating soundscape emotional constructs, the ICSAQ scale emerges as a complementary tool to Western instruments, developed through a process of identifying culturally grounded structures and items. The Chinese cultural context offers new perspectives for identifying commonalities in human soundscape affect as well as differences caused by cultural variables, with particular emphasis on identifying indigenous SAQ structures and generating culturally relevant items. These steps in the testing process provide cross-cultural psychologists with a valuable framework for developing culturally appropriate indigenous SAD inventories. To generalise the findings of the SAQ model and the ICSAQ scale, further research is needed on these models and possibly to compare their validity in predicting criterion variables. These criterion variables may include short-term reactions and emotion, as well as behaviour.

The concepts of native and imported structures are used in a continuum rather than a dichotomy^69^, cross-cultural and native SAQ research goes hand in hand, and a comprehensive study of soundscape emotion should accommodate both. To expand the understanding of universal soundscape emotion, a holistic perspective needs to be adopted. Soundscape research should define the universality and cultural specificity of soundscape emotion^55^ to accommodate different cultures and develop culturally relevant instruments accordingly^70^. In this context, the combined emic-etic approach provides an effective perspective^71^. This includes not only existing SAQ assessment methods in the West but also the incorporation of folk-emotional concepts from specific cultures and the experiences of professionals. Our study revealed that soundscape emotion reflects both the commonalities across cultures and the uniqueness of Chinese soundscape emotion.

In the present study, we found biases in the imported SAQ scale in measuring the ICSAQ scale, raising concerns about the validity and accuracy of affective quality scales in cross-cultural research. Research suggests that emotional experience varies systematically across cultural groups^10,72^. The bias stem from culturally contextualised affective sematic space differences, with sounds having different symbolic meanings and affective associations in different cultures. Therefore, the use of scales constructed by other cultures in cross-cultural contexts may not accurately capture the true responses to the SAQ of individuals in different cultural contexts and may easily result in cultural stereotyping. The same bias problem may also occur in the measurement of other forms of environmental affective quality, where the emotional experience attributed to the environment is also deeply rooted in the cultural environment and influenced by cultural values and social practices. The untranslatability of the affective quality scale in cross-cultural measurement refers not only to the problem of language translation but also, more importantly, to differences in cultural context. In there, geographical proximity factors could partly explain these differences. To assess environmental affective quality effectively in different cultural contexts, it may be necessary to adapt scale items culturally or design culturally specific scales to verify the reliability and validity of cross-cultural scales. This means that, in addition to considering domain-specific emotional constructs, culturally specific aspects need to be included to ensure the scientific application of scales in multicultural contexts. Ultimately, our findings underline the necessity of culturally sensitive and contextually validated affective assessment tools, enabling more accurate evaluation and more inclusive approaches in environmental management and urban planning.

## Methods

### Outline

The research was conducted in three stages. In the first stage, 424 soundscape excerpts (SEs) were recorded across 17 cities in China using binaural acoustic recording and panoramic photography. From these recordings, 132 SEs were selected based on criteria such as duration, representativeness, and diversity of sound events. The selected SEs were analysed for acoustic parameters, and their dominant sound categories were assessed.

The second stage focused on developing Chinese soundscape affective descriptors (SADs). The “Soundscape Collection and Recording” project was conducted, obtaining 2,096 soundscape descriptions from 23 university student participants. SADs were extracted from these texts and combined with translated English SADs, resulting in a total of 449 descriptors. Through an expert focus group and an online appropriateness experiment,108 Chinese SADs were determined.

The third stage involved a soundscape perception experiment. 264 university student participants from 30 Chinese provinces rated the 108 SADs for 132 SEs in a laboratory listening experiment. Using the complete dataset of ratings, the SAQ model was developed through PCA. The stability of the SAQ structure was validated using Clustering-PCA in sub-datasets. Finally, based on the soundscape semantic space, the Chinese SAQ scale was developed.

### Soundscape excerpts acquisition and screening

To comprehensively cover the soundscape of different regions in China, 17 cities in 13 provinces of China were selected for soundscape recording, taking into account the principles and strategies of geographic distribution (e.g., south China, north China, east China, etc.), city size (e.g., megacities, type I large cities, medium-sized cities, etc.) and type of natural geographic conditions (e.g., mountainous, basin; bay type, etc.). The cities include Jinan, Xiamen, Wenzhou, Wuhan, Chongqing, Zibo, Changshu, Shenyang, Tianjin, Urumqi, Hangzhou, Harbin, Yining, Duyun, Kunming, Taiyuan, and Lvliang. time periods, 2023.5.2-2023.5.4 and 2023.9.27-2023.10.15 and 2024.8.7-2024.8.20. Binaural audio capture was performed using the SQope device (high-fidelity binaural recording) in combination with the HEAD B2U application to capture the human ear as it is heard in the environment. At the same time, panoramic shots were taken using a GoProMAX camera to record the context corresponding to the acoustic environment at the moment. A total of 424 SEs were captured.

To ensure that the captured audio is accepted as a valid SE and broadly encompasses urban spatial types, the following conditions must be met: 30 s of Pure Audio^15^; the SE must accurately represent the specific location in which the recording takes place; the SE must include not only the field context but also multiple sound events (“soundscape” and “sound event”), and the inclusion of only a single foreground sound (e.g., a car or a pedestrian passing by) should not be considered a complete soundscape because of the lack of an appropriate acoustic context. A final selection of 132 SEs was made from 11 different spatial types: urban parks (40), suburban parks (11), city streets (32), city squares (23), sports areas (1), transport hubs (5), residential areas (3), playgrounds (3), courtyards (4), waterfronts (8) and campuses (2).

### Basic acoustic analysis of the acquired soundscape excerpts

A total of 132 SEs were extracted and analysed for physical acoustic and psychoacoustic parameters (based on the average sound pressure levels of the left and right channels of the binaurally recorded soundscape clips) using B&K Connect acoustic analysis technology. A variety of acoustic metrics were covered, using the 30-second equivalent continuous sound level (*L*_Aeq,30s_ or *N*_5_) as a measure of the overall loudness of the soundscape; *L*_A5_ *- L*_A95_ or *N*_5_*/N*_95_ to represent the range of variation in sound intensity in the soundscape; and *L*_(C)_ *-L*_(A)_ to represent the relative proportions of low-frequency sounds. In addition, several psychoacoustic parameters were used to quantify the perceived quality of sound: roughness (*R*), sharpness (*S*), tonality (*T*), and fluctuation (*F*). See ISO-12913-3 for methodological calculations of these parameters. Supplementary Information Table S2 shows descriptive statistics for the acoustic variables of the 132 SEs.

SEs cover a rich variety of environmental sound types, such as traffic sounds (aircraft, personal cars, motorbikes, car alarms and car horns); natural sounds (sounds such as birdsong, wind ruffling the grass, leaf rubbing, running water, fountains, waterfalls, etc.); human sounds (including the sounds of children’s playfulness, footsteps, and other human activity sounds); and mechanical sounds (sounds generated by machine operations such as chainsaws, rock drills, and sweeping arcade machines). SE’s dominant sound category was assessed via the jury test^73^, and “dominant” sound sources were defined as a category of sound that occupies a significant proportion of the 30-second clip and considered to be a foreground sound. Three members of the research team independently listened to all SEs, which were coded and synthesised to reach a unified result. Four dichotomous “dominant sound category” variables (traffic, mechanical, natural and human sounds) were coded for each SE to distinguish dominant from nondominant sound sources. To eliminate assessment bias, the evaluators were unaware of the exact source of the recordings throughout the process, and the SEs were played in random order.

The overall sound pressure levels across the 132 SE samples displayed a normal distribution. To validate the robustness of the SAQ structure, sub-datasets were established based on the regional distribution and urban spatial types. The two pairs of sub-datasets are: North (*N* = 67) and South (*N* = 65); artificial (*N* = 61) and natural spaces (*N* = 71) (Descriptive statistics of the acoustic variables are shown in Supplementary Information Table S2 through S6). The *L*_Aeq_ of North space ranged from 44.6 to 77.7 dB (mean = 61.9 dB, SD = ± 6.8), whereas that of south space ranged from 45.2 to 75.8 dB (mean = 59.9 dB, SD = ± 8.3), no statistically significant difference in *L*_Aeq_ between the two spaces (*F* = 2.460, *p* = 0.119 > 0.05). The *L*_Aeq_ of natural space ranged from 44.6 to 70.5 dB (mean = 57.9 dB, SD = ± 6.5), whereas that of artificial space ranged from 46.3 to 77.7 dB (mean = 63.4 dB, SD = ± 7.6), there is a statistically significant difference in *L*_Aeq_ between the two spaces (*F* = 19.903, *p* = 0.000 < 0.05). Through one-way ANOVA (Assuming the normality and homogeneity of variance), significant differences (*p* < 0.05) were found between natural space and artificial space in several acoustic metrics (e.g., *L*_Aeq_, *L*_A5_, *L*_A95_, *L*_C_, *N*_5_, *N*_95_, *S*_5_, *S*_95_ and *R*_10_, *R*_50_) (Supplementary Information Table S7, S8).

### Gathering of indigenous Chinese SADs

To obtain local descriptions of soundscape emotions, this study conducted a soundscape collection and recording project to systematically collect and analyse descriptive data on soundscape experiences. The Soundscape Collection and Recording project was conducted to systematically collect and analyse descriptive data on soundscape experiences. A total of 23 university student participants were recruited for the project, and each participant was asked to record ten pieces of soundscape per day via a designated device during the ten-day survey period; to record the environment, time, location, and type of sound source; and to provide a description of their soundscape perceptions and experiences. A total of 2300 audio samples were recorded throughout the course of the project, and after screening, 2096 valid audios were eventually included in the study. Through text analysis of the verbal descriptions of these valid audios, 206 Chinese adjectives commonly used to describe soundscapes in everyday natural language were extracted. These adjectives were labelled to identify single or multiple soundscape features. Given that the interviews were highly structured, it was appropriate for a single researcher to carry out qualitative data collection and initial analysis^74^.

A list of 116 English adjectives describing the SAQ, as listed by Axelsson et al.^19^, was introduced as a supplement to this process. A systematic translation strategy was adopted to incorporate the adjectives into this study. First, preliminary translations were carried out with the help of an online dictionary tool as well as by referring to authoritative English–Chinese dictionaries. As many English adjectives may have multiple counterparts in Chinese, even some words do not have directly corresponding Chinese expressions. Through contextual analyses and expert discussions, specific expression habits in the Chinese language and culture were also considered. Finally, 374 Chinese adjectives were extracted. Since the applicability of the translation results cannot be determined by the translation itself alone, further testing and verification are needed.

The list of extracted adjectives was filtered. The total number of adjectives was 449, of which 29% were duplicates. The screening process involved five experts in the field of soundscape, prioritising adjectives that describe the affect (feeling) towards the soundscape and reflect the characteristics of the soundscape. Words such as “old” were excluded because of their associated perceptual/cognitive meaning, despite their emotional connotations. In addition, adjectives such as “fearful” were excluded because they were only applicable to a specific soundscape and lacked general applicability to a wide range of urban public space soundscapes. Adjectives describing only the physical properties of sound, e.g., “loud”, were also excluded. In the end, 171 soundscape affective adjectives were retained.

### SAD filtering via online screening experiment

A remote listening experiment in which a total of 29 evaluators from the fields of soundscape (20), human settlement (4), and linguistics and sociology (5) were recruited to participate in the online experiment was conducted. All the evaluators were hearing impaired and had no other significant health problems to ensure that they were able to accurately participate and complete the experimental tasks. The experiment strictly adhered to the relevant ethical norms, and all the evaluators signed an informed consent form before the start of the experiment and were informed in detail about the purpose and procedure of the experiment. To protect the privacy of the evaluators, the anonymity and confidentiality of all the data were ensured. The experiment was conducted through Gorilla Experiment Builder (www.gorilla.sc), a professional online experimental platform dedicated to the design and hosting of complex behavioural studies. The survey was distributed to pre-registered evaluators through Prolific (www.prolific.co). The data collection period was from 25 June 2023 to 4 July 2023. The online data collection tool was active at all times during this period to ensure that the target number of participants was reached. The audio stimuli used for the experiment consisted of eight 30-second binaural recordings (24-bit, 48 kHz, stereo WAV files) recorded in urban open spaces during the summer and autumn of 2019. These SEs cover a wide range of spatial types, including parks, roads, construction sites, waters, green spaces, dense forests and squares. A wide range of sound elements can be recognised, such as traffic, construction, high-pressure water jets, streams, birdsong, conversations, footsteps, children playing and street performances.

The average length of an experimental session was 60 min, including instructions on the process of receiving the experiment and necessary breaks. To prevent fatigue, appropriate breaks were scheduled during the experimental session to enable the evaluators to perform at their best. Consistency in the testing environment and equipment setup was ensured. First, evaluators were required to wear wired in-ear headphones without noise cancellation to ensure that the original quality of the audio stimuli was not disturbed. Prior to the start of the experiment, the evaluator adjusted the Sound Volume Setting of the computer by playing a specific audio stimulus (white noise, 60 s, 26 dB). The evaluator subsequently would listen to all the SEs and judge the appropriateness of adjectives for describing the soundscape in the context of his/her own personal experience and experience in daily life. The adjectives were presented sequentially on the computer screen in random order, and the evaluator assessed the appropriateness of each adjective using a Likert scale ranging from 1 (“very unsuitable”) to 5 (“very suitable”). This single-measurement setup helped reduce possible order effects during the evaluation process. A total of 108 emotional words suitable for describing soundscapes, defined as SADs, were analysed by frequency and standard deviation analyses, of which 70 SADs came from the “Soundscape Collection and Recording” project. The explanation of meaning for the 108 Chinese SADs can be found in Supplementary Information Table S9.

### Participants of the soundscape perception experiment

A total of 264 university student participants were recruited for this Chinese experiment (separate from the 23 participants from the second phase), including 127 males and 137 females, with a mean age of 21.0 years (age range 18–27 years). These participants came from 30 provinces in China, with extensive regional coverage (Fig. 2c). And all the participants have lived in China for a long time and have no long-term living experience abroad. The geographical area in which an individual grows up significantly influences his or her evaluation of the soundscape^15^. Cultural backgrounds, ambient noise levels, and daily habits in different geographic areas shape people’s perceptions of and responses to the soundscape. Therefore, generalisability and external validity were improved by recruiting participants from different provinces. All participants were rigorously screened to confirm that they were free of hearing impairment and did not suffer from other major health problems. Prior to participating in this study, all the participants provided written self-reports on their sex, gender and place of origin along with informed consent and were informed in detail about the experimental procedures and purpose. All procedures of this study strictly followed the relevant ethical norms and were approved by the Ethics Committee of Shenyang Architecture University.

### Experimental procedure

The experiments were conducted in a semi-echoic listening laboratory (Specific location: Shenyang Jianzhu University, Shenyang, China) via a desktop computer equipped with BK connect software to control the sound playback. Sound output was achieved using an external sound card (Headphone Amplifier ZE 0769) and a Sennheiser HD 650. To reproduce the acoustic environment in the SE, sound calibration was performed using a multimeter (FLUKE15B+ Digital) and a calibration signal (1 kHz sinusoidal signal SPL 94 dB recorded). In addition, the background noise in the laboratory was monitored during the experiment using a sound level meter (Optimus cr:160), which was controlled at 15.5 dB(A) to ensure that the experiment was free from external interference.

To keep the experimental time within reasonable limits, each participant assessed only 6 of the 132 SEs. Evaluations were performed using an irregular selection procedure, where each SE was evaluated by 12 different participants. A scale containing 116 SADs (108 SADs developed here and 8 SADs translated from the TGSAQ scale) was provided to measure the “SAD-soundscape match” for each SE, each SAD was equipped with a 100 mm visual analogue scale, and the end-points were marked as “No match at all (0%)” and “Perfect Match (100%).” Data was collected through the online survey platform www.lediaocha.com. Images of the tool’s user interface can be found in Fig. S1. The participants were instructed to draw vertical lines on 6 SEs listened to assess how well the SAD matched their soundscape perception by drawing vertical lines on the 116-attribute scale. To avoid order effects, these SAD scales were randomly organised into 1584 different orders. The assignment of SEs and SAD scales followed three principles: a) to ensure that the same SE was not evaluated twice using the same order of the SAD scales; b) to avoid a single participant evaluating the same order of the SAD scales twice; and c) not to repeat playing the SEs in the same order as the participants. Before starting the formal experiment, the participants were required to perform the evaluation exercise (additional excerpts). The experiment was conducted in groups of six, each group of participants was asked to listen to six different SEs, and the six SEs were repeated four times in different presentation orders. After each playback, the participants were asked to rate the soundscape they heard on a set of attribute scales. The average length of the entire experimental session was 60 min, which included the playback test of the SEs, the experimental instructions, and a break in between.

### Statistical analyses

Principal component analysis (PCA) is a multivariate statistical method that combines information from several variables observed on the same subject into fewer variables, called principal components (PCs)^75^. PCs have geometric properties that allow intuitive and structured interpretation of the soundscape affective qualities in the main features inherent for intuitive and structured interpretation, revealing the intrinsic connections and logical structure between SADs. For each of the 132 SEs, the arithmetic mean of the 12 participants’ ratings of each SAD was computed to minimise the effects of measurement error as well as individual differences, resulting in a 132 × 108 data matrix that was subjected to PCA. To assess the reliability of the PCA results, 90 samples were randomly sampled from the 132 samples to create datasets A and B. The results revealed that the samples demonstrated stable and nearly consistent patterns and structures regardless of how they were partitioned. The factor loadings and variance explained for each PC were similar in datasets A and B. For PC1, 2 Pearson’s coefficients were 0.98 and 0.98 (*p* < 0.01), respectively.

Clustering analysis helps to understand and categorise the relationships of SADs. Hierarchical clustering analysis and K-means clustering analysis were carried out on 108 SADs. First, the most similar SAD pairs were gradually merged by the hierarchical clustering analysis, the most similar SAD pairs were gradually merged, and the resulting dendrogram produced 16 highly homogeneous clusters (Supplementary Information Table S10). Then, on the basis of the results of hierarchical clustering analysis, the SADs are assigned to the predefined 16 clusters through an iterative optimisation process of K-means clustering analysis, where 7 SADs are excluded from these 108 attributes owing to their specificity or low similarity with other SADs. Distance in cluster analysis was used as a correlation between the 108 attributes^76^. PCA was performed on these 16 SAD clusters in all categorical datasets to explore the distribution and similarity of these attributes to further clarify the SAQ dimensions.

Using the ICSAQ scale, the affective quality can be attributed to a single point in the space shown in Fig. 3a, thus quantitatively measuring the effect of a specific soundscape. This point can be represented by the values of the x-coordinate and y-coordinate, which are calculated using the following equations:

The equivalent equations for the TGSAQ scale are included in ISO 12913-3.

Next, a multiple regression model was used to assess the interpretability of each dimension. First, a correlation analysis was conducted to screen for the acoustic indicators that were highly correlated with the dependent variable and eliminate indicators with strong collinearity to ensure the stability and accuracy of the model. In the Comfort or Pleasantness model, *L*_Aeq_ was included as the main indicator, combined with the categorical variables of natural sounds, human voices, traffic sounds and mechanical sounds being dominant. For the Richness or Eventfulness model, *L*_(C)_ *- L*_(A)_ was included, again combined with variables for the dominant sound sources.

## Supplementary information

Supplementary information

## Data Availability

All data supporting the findings of this study are available within the article and the Supplementary Information. Reasonable request for additional information can be directed to corresponding authors.

## Abbreviations

SAD: soundscape affective descriptor
SAQ: soundscape affective quality
SEs: soundscape excerpts
ICSAQ scale: Indigenous Chinese SAQ scale
TGSAQ scale: translated global SAQ scale
PCA: principal component analysis
PC: principal component
