# Supplementary material for: The untranslatability of environmental affective scales: insights from indigenous soundscape perceptions in China
Source: NPJ Urban Sustain. 2025 Jun 14;5(1):38. doi: 10.1038/s42949-025-00228-6 (PMC12167170; doi:10.1038/s42949-025-00228-6)
Supplement: Supplementary file 1 — Supplementary information [file 42949_2025_228_MOESM1_ESM.pdf]

Supplementary Information

The untranslatability of Environmental Affective Scales: Insights from indigenous soundscape perceptions in China

Duotuo Wu[a,b], Rumei Han[a,c], Ruining Zhang[a,d], Xinhao Yang[a,b], Yuan Zhang[a,b,*], Jian Kang[e*]

[a] Liaoning Provincial Key Laboratory of Eco-Building Physics Technology and Evaluation, Shenyang, China; [b] School of Architecture and Urban Planning, Shenyang Jianzhu University, Shenyang, China; [c] School of Science, Shenyang Jianzhu University, Shenyang, China; [d] School of Architecture, Tianjin University, Tianjin, China; [e] UCL Institute for Environmental Design and Engineering, The Bartlett, University College London, London, United Kingdom. *Correspondence: Yuan Zhang (y.zhang@sjzu.edu.cn), Jian Kang (j.kang@ucl.ac.uk).

Table S1 Reliabilities and intercorrelations among Statistics for the Four Bipolar Scales of China Affective Quality

Scale	No. of items	M	SD	a (Cronbach Alpha)	Correlation among scales		
					2	3	4
1. shushide - hunluande	2	-10.5	25.8	0.84	.491**	.081	.623**
2. pingdande - renaode	2	-16.3	22.6	0.70	-	-.628**	-.170
3. fengfude - dandiaode	2	23.3	21.0	0.76	-	-	.646**
4. youqude - wuliaode	2	3.3	22.8	0.84	-	-	-

The statistics are based on a sample of 132.

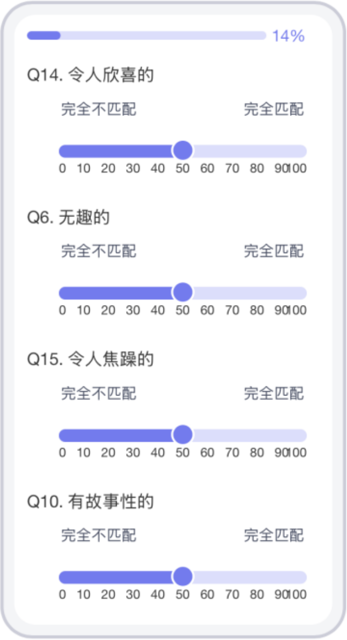

Figure S1 100 mm visual analogue scale: Image of the operation interface of the tool used by participants in the soundscape perception experiment

Table S2 Descriptive statistics of the acoustic variables calculated for all **132** soundscape excerpts.

Acoustic variables	N	Min	Max	Mean	SD	Median
L_{Aeq}	132	44.62	77.72	60.87	7.61	60.53
L_{A5}	132	47.34	81.23	64.32	7.97	63.39
L_{A95}	132	39.85	73.81	56.54	7.27	56.76
L_{A5} - L_{A95}	132	1.77	19.05	7.78	3.50	7.33
N_5	132	5.12	46.05	18.24	8.90	16.25
N_{95}	132	3.01	32.60	11.63	5.55	10.45
N_5/N_{95}	132	1.16	2.84	1.58	0.30	1.52
S_5	132	1.05	3.43	1.54	0.30	1.51
S_{95}	132	0.79	2.97	1.17	0.24	1.16
R_{10}	132	1.74	3.34	2.21	0.25	2.15
R_{50}	132	1.44	2.26	1.80	0.15	1.78
F_{10}	132	1.59	4.64	2.90	0.63	2.95
F_{50}	132	1.16	3.58	2.17	0.55	2.16
$L_{(C)}$	132	52.33	84.29	67.56	6.68	66.98
$L_{(C)}$ - $L_{(A)}$	132	-1.18	16.27	6.68	3.61	6.14

Table S3 Descriptive statistics of the acoustic variables calculated for all the **61** soundscape excerpts from **natural spaces**.

**Acoustic variables**	**N**	**Min**	**Max**	**Mean**	**SD**	**Median**
L_{Aeq}	61	44.62	70.45	57.89	6.45	57.75
L_{A5}	61	47.34	72.74	61.23	6.66	60.65
L_{A95}	61	40.35	68.29	53.76	6.30	53.55
L_{A5} - L_{A95}	61	2.13	18.00	7.47	3.10	7.27
N_5	61	5.12	31.05	14.56	6.23	13.30
N_{95}	61	3.44	22.55	9.47	4.09	8.86
N_5/N_{95}	61	1.21	2.42	1.56	0.25	1.52
S_5	61	1.17	3.43	1.62	0.34	1.56
S_{95}	61	0.86	2.97	1.23	0.29	1.17
R_{10}	61	1.74	3.14	2.22	0.25	2.16
R_{50}	61	1.44	2.22	1.80	0.14	1.78
F_{10}	61	1.62	4.30	2.90	0.65	3.03
F_{50}	61	1.31	3.56	2.25	0.56	2.28
$L_{(C)}$	61	52.33	77.27	64.46	5.30	64.68
$L_{(C)}$ - $L_{(A)}$	61	-1.18	13.24	6.56	2.94	6.53

Table S4 Descriptive statistics of the acoustic variables calculated for all **71** soundscape excerpts from **artificial spaces**.

Acoustic variables	N	Min	Max	Mean	SD	Median
L_{Aeq}	71	46.31	77.72	63.44	7.64	62.65
L_{A5}	71	50.82	81.23	66.97	8.09	65.94
L_{A95}	71	39.85	73.81	58.93	7.23	59.27
L_{A5} - L_{A95}	71	1.77	19.05	8.04	3.82	7.33
N_5	71	6.76	46.05	21.41	9.65	18.15
N_{95}	71	3.01	32.60	13.49	5.97	12.65
N_5/N_{95}	71	1.16	2.84	1.61	0.33	1.53
S_5	71	1.05	2.12	1.47	0.23	1.46
S_{95}	71	0.79	1.54	1.13	0.17	1.15
R_{10}	71	1.82	3.34	2.20	0.25	2.14
R_{50}	71	1.52	2.26	1.81	0.15	1.78
F_{10}	71	1.59	4.64	2.90	0.61	2.89
F_{50}	71	1.16	3.58	2.11	0.53	2.05
$L_{(C)}$	71	55.62	84.29	70.22	6.63	71.66
$L_{(C)}$ - $L_{(A)}$	71	0.57	16.27	6.78	4.12	5.57

Table S5 Descriptive statistics of the acoustic variables calculated for all **67** soundscape excerpts from **North spaces**.

Acoustic variables	N	Min	Max	Mean	SD	Median
L_{Aeq}	67	44.62	77.72	61.89	6.76	61.19
L_{A5}	67	47.34	81.23	65.31	7.23	63.66
L_{A95}	67	40.87	73.81	57.77	6.38	58.32
$L_{A5} - L_{A95}$	67	2.13	18.00	7.54	3.28	7.18
N_5	67	5.12	46.05	19.28	8.73	16.90
N_{95}	67	3.44	32.60	12.50	5.43	11.75
N_5/N_{95}	67	1.20	2.42	1.55	0.25	1.49
S_5	67	1.05	2.05	1.50	0.25	1.48
S_{95}	67	0.84	1.54	1.13	0.17	1.14
R_{10}	67	1.74	3.14	2.20	0.25	2.13
R_{50}	67	1.44	2.26	1.78	0.15	1.76
F_{10}	67	1.59	4.64	2.86	0.62	2.92
F_{50}	67	1.16	3.56	2.15	0.53	2.14
$L_{(C)}$	67	55.78	81.99	69.29	5.78	69.17
$L_{(C)} - L_{(A)}$	67	0.57	14.81	7.39	3.73	6.85

Table S6 Descriptive statistics of the acoustic variables calculated for all **65** soundscape excerpts from **South spaces**.

Acoustic variables	N	Min	Max	Mean	SD	Median
L_{Aeq}	65	45.24	75.87	59.83	8.33	59.41
L_{A5}	65	48.28	81.02	63.30	8.61	63.01
L_{A95}	65	39.85	69.38	55.28	7.94	55.41
L_{A5} - L_{A95}	65	1.77	19.05	8.02	3.72	7.44
N_5	65	5.79	36.72	17.18	9.02	14.90
N_{95}	65	3.01	22.44	10.74	5.58	9.96
N_5/N_{95}	65	1.16	2.84	1.62	0.34	1.55
S_5	65	1.08	3.43	1.58	0.34	1.55
S_{95}	65	0.79	2.97	1.22	0.29	1.18
R_{10}	65	1.93	3.34	2.23	0.26	2.16
R_{50}	65	1.52	2.25	1.83	0.13	1.81
F_{10}	65	1.62	4.15	2.95	0.63	3.05
F_{50}	65	1.28	3.58	2.20	0.57	2.24
$L_{(C)}$	65	52.33	84.29	65.77	7.11	65.29
$L_{(C)}$ - $L_{(A)}$	65	-1.18	16.27	5.95	3.36	5.27

Table S7 Statistical Analysis of Soundscape Differences between Natural and Artificial space in China

Acoustic variables	Sum of Squares (SS)	Degrees of Freedom (df)	Mean Square (MS)	F-value	p-value
L_{Aeq}	1008.2	1	1008.2	19.903	0.000***
L_{A5}	1082.1	1	1082.1	19.414	0.000***
L_{A95}	876.6	1	876.6	18.856	0.000***
L_{A5} - L_{A5}	10.8	1	10.8	0.881	0.350
N_5	1540.9	1	1540.9	22.660	0.000***
N_{95}	531.8	1	531.8	19.731	0.000***
N_5/N_{95}	0.1	1	0.1	0.881	0.350
S_5	0.7	1	0.7	7.774	0.006**
S_{95}	0.3	1	0.3	6.337	0.013*
R_{10}	0	1	0	0.376	0.541
R_{50}	0	1	0	0.125	0.724
F_{10}	0	1	0	0.001	0.975
F_{50}	0.6	1	0.6	2.139	0.146
$L_{(C)}$	1089.9	1	1089.9	29.740	0.000***
$L_{(C)}$ - $L_{(A)}$	1.6	1	1.6	0.121	0.728

*p < 0.05, **p < 0.01, ***p < 0.001

Table S8 Statistical Analysis of Soundscape Differences between South and North space in China

Acoustic variables	Sum of Squares (SS)	Degrees of Freedom (df)	Mean Square (MS)	F-value	p-value
L_{Aeq}	141	1	141	2.460	0.119
L_{A5}	132.7	1	132.7	2.105	0.149
L_{A95}	204	1	204	3.949	0.049
L_{A5} - L_{A95}	7.6	1	7.6	0.621	0.432
N_5	146.2	1	146.2	1.857	0.175
N_{95}	102.3	1	102.3	3.383	0.068
N_5/N_{95}	0.2	1	0.2	1.999	0.16
S_5	0.2	1	0.2	2.001	0.16
S_{95}	0.2	1	0.2	3.902	0.05*
R_{10}	0	1	0	0.511	0.476
R_{50}	0.1	1	0.1	3.660	0.058
F_{10}	0.3	1	0.3	0.689	0.408
F_{50}	0.1	1	0.1	0.259	0.612
$L_{(C)}$	406.9	1	406.9	9.712	0.002**
$L_{(C)}$ - $L_{(A)}$	68.8	1	68.8	5.461	0.021

*p < 0.05, **p < 0.01, ***p < 0.001

Table S9 These are word explanations of 108 Chinese SADs from the Cambridge Dictionary. They are for reference only and do not constitute complete definitions or explanations.

Number	Affective State	Zhuyin Fuhao (Bopomofo)	Word explanation
1	生动的	Shengdongde	1.Vivid descriptions, memories, etc. produce very clear, powerful, and detailed images in the mind<br>2.very clear and powerful
2	有氛围感的	Youfenweigande	X
3	有画面感的	Youhuamiangande	X
4	令人振奋的	Lingrenzhenfende	1.used to describe something that encourages you and makes you feel happier<br>2.making you feel happier and more positive<br>3.making someone feel better<br>4.in a way that makes you remember or imagine something pleasant
5	丰富的	fengfude	1.larger than usual or expected<br>2.more than enough, If something is plentiful, there is a lot of it available
6	热闹的	Renaode	1.A busy place is full of activity or people<br>2.If a place is bustling, it is full of busy activity<br>3.very loud and uncontrolled, and full of energy
7	令人欣喜的	Lingrenxinxide	beautiful or interesting in a way that means you cannot stop listening to or watching someone or something
8	生气勃勃的	Shengqibobode	1.full of energy and enthusiasm; interesting and exciting<br>2.having a lot of energy or activity
9	喜庆的	Xiqingde	having or producing happy and enjoyable feelings suitable for a festival or other special occasion
10	引发联想的	Yinfalianxiangde	X
11	令人兴奋的	Lingrenxingfende	to make someone excited and interested about something
12	有年代感的	Youniandaigande	X
13	有生机的	Youshengjide	in a way that is able to continue to exist or develop into a living being
14	有提示性的	Youtishixingde	X
15	有生活气息的	Youshenghuoqixide	X
16	有趣的	Youqude	1.used to describe a situation or activity that gives you great pleasure
17	令人激动的	Lingrenjidongde	1.to make someone have strong feelings of happiness and enthusiasm<br>2.to make yourself or another person feel upset or feel strong emotions
18	富有变化的	Fuyoubianhuade	X
19	令人开心的	Lingrenkaixinde	If something tickles you, you find it funny or it makes you happy
20	富有情感的	Fuyouqinggande	X
21	空旷的	Kongkuangde	1.If a place is deserted, there are no people in it<br>2.A forlorn place feels empty

22	幸福的	Xingfude	1.feeling, showing, or causing pleasure or satisfaction
23	引起回忆的	Yinqihuiyide	1.If something takes you back, it makes you remember a period or an event<br>2.making you remember or imagine something pleasant
24	温和的	Wenhede	1.quiet and easy to influence, persuade, or control<br>2.gentle and calm
25	有活力的	Youhuolide	1.having or involving a lot of energy
26	熟悉的	Shuxide	1.easy to recognize because of being seen, met, heard, etc. before<br>2.able to understand or recognize something
27	令人愉快的	Lingrenyukuaide	1.pleasant, enjoyable, or satisfactory<br>2.happy or showing enjoyment
28	令人欢乐的	Lingrenhuanlede	friendly and making you feel happy and welcome
29	引人注意的	Yinrenzhuyide	1.very attractive in a way that attracts a lot of attention<br>2.of an unusual quality or standard; noticeable
30	嘈杂的	Caozade	1.making a lot of noise<br>2.filled with noise and activity
31	令人不舒服的	Lingrenbushufude	1.If something gets to you, it makes you suffer<br>2.in a way that makes you feel slightly embarrassed, or that shows that you feel slightly embarrassed
32	令人紧张的	Lingrenjinzhangde	1.to make someone feel less confident and slightly frightened<br>2.nervous or frightened, often in a way that is not reasonable
33	令人厌烦的	Lingrenyanfande	1.annoyed and disappointed with something or someone<br>2.used to describe a situation that is confused and unpleasant<br>3.an activity or situation that is annoying or unpleasant
34	喧嚣的	Xuanxiaode	1.loud and powerful<br>2.extremely noisy and confused
35	有压迫感的	Youyapogande	X
36	喧闹的	Xuannaode	1.noisy, energetic, and rough<br>2.very loud and uncontrolled, and full of energy
37	令人讨厌的	lingrentaoyande	used to emphasize that you find something unpleasant or annoying
38	烦人的	Fanrende	1.not interesting or exciting<br>2.very bad or unpleasant
39	恼人的	Naorende	1.making you feel annoyed<br>2.unpleasant and unattractive
40	难听的	Nantingde	1.A rough voice or sound is hard and loud<br>2.unpleasant to listen to
41	混乱的	Hunluande	1.in a confused or badly organized state<br>2.in a state of chaos
42	令人烦恼的	Lingrennaofande	1.to make someone unhappy and angry<br>2.When a noise or behaviour grates, it annoys you

43	令人不适的	Lingrenbushide	1.worried and uncomfortable<br>2.If you have a tight feeling in your chest you have an uncomfortable feeling of pressure, caused by illness, fear, etc
44	吵闹的	Chaonaode	very loud, or full of confusion, change, or uncertainty
45	有韵律的	Youyunlvde	X
46	有故事性的	Yougushixingde	X
47	令人烦躁的	Lingrenfanzaode	When a noise or behaviour grates, it annoys you
48	引人遐想的	Yinrenxiaxiangde	X
49	令人焦躁的	Lingrenjiaozaode	1.nervous and worried and unable to relax<br>2.nervous or confused because you are worried about something
50	有干扰性的	Youganraoxingde	X
51	身临其境的	Shenlinqijingde	X
52	令人困倦的	Lingrenkunjuande	in a way that shows you are tired and want to sleep
53	令人不快的	Lingrenbukuaide	1.bad or very unpleasant<br>2.not enjoyable or pleasant
54	无趣的	Wuqude	1.not interesting or completely without imagination<br>2.used to suggest that something is boring and has no excitement
55	寂静的	Jijingde	without any sound
56	沉闷的	Chenmende	A place is not attractive and contains nothing interesting or pleasant
57	压抑的	Yayide	1.a feeling of being very uncomfortable and worried<br>2.to not allow something, especially feelings, to be expressed
58	枯燥的	Kuzaode	1.not interesting or exciting in any way<br>2.not interesting and showing no imagination
59	无变化的	Wubianhuade	continuous or regular
60	令人反感的	Lingrenfangande	1.feeling extreme dislike or disapproval of something<br>2.making someone feel a strong dislike for something, or making them not want to do it
61	冷清的	Lengqingde	showing little activity; not busy or happening in a positive way
62	平淡无奇的	Pingdanwuqide	1.An uneventful time or situation is one in which nothing interesting or surprising happens<br>2.a characterless person or thing is not interesting or has no style or unusual qualities
63	平淡的	Pingdande	1.no new ideas or too often repeated, and therefore not funny or interesting<br>2.without excitement, or not very noticeable
64	无聊的	Wuliaode	1.showing no interest or mental activity<br>2.in a way that is extremely silly or has no real meaning or importance
65	凄凉的	qiliangde	If a place is bleak, it is empty, and not welcoming or attractive
66	沉寂的	Chenjide	If a place is dead, it is too quiet and nothing interesting happens there
67	令人抓狂的	Lingrenzhuakuangde	X

68	无体验感的	Wutiyangande	X
69	糟糕的	Zaogaode	1.very unpleasant or serious or of low quality<br>2.very bad, of very low quality, or shocking and very sad
70	奇怪的	Qiguaide	strange, surprising, unexpected, or difficult to explain or understand
71	令人抵触的	Lingrendichude	a feeling of disgust caused by behaviour or beliefs, etc. which are very unpleasant
72	令人沮丧的	Lingrenjusangde	1.feeling or showing sadness<br>2.unhappy, disappointed, or without hope
73	乏味的	Faweide	1.not having a strong taste or character, or having no interest or energy<br>2.showing little energy or interest
74	舒缓的	Shuhuande	slow and relaxed
75	亲切的	Qinqiede	the quality of being pleasant and friendly
76	空灵的	Konglingde	X
77	宁静的	Ningjingde	1.having little activity or excitement and few people<br>2.having a calm appearance or characteristics
78	吸引人的	Xinyinrende	X
79	放松的	Fangsongde	1.feeling happy and comfortable because nothing is worrying you<br>2.relaxed, not worrying about anything
80	静谧的	Jingmide	X
81	悦耳的	Yueerde	If a sound is sweet, it is pleasant and easy to like
82	柔和的	Rouhede	1.not forceful, loud, or easily noticed<br>2.pleasantly soft or light
83	平和的	Pingdande	1.having a calm appearance or characteristics<br>2.not violent, severe, or strong
84	恬静的	Tianjingde	An idyllic place or experience is extremely pleasant, beautiful, or peaceful
85	有吸引力的	Youxiyinlide	very attractive; as good as it could be
86	有感染力的	Youganranlide	Something that is infectious has an effect on everyone who is present and makes them want to join in
87	优美的	Youmeide	1.moving in a smooth, relaxed, attractive way, or having a smooth, attractive shape
88	安静的	Anjingde	1.calm and peaceful and without noise, violence, worry, etc<br>2.avoiding excitement or great activity and usually calm and relaxed
89	动听的	Dongtingde	If a sound is sweet, it is pleasant and easy to like
90	单调的	Dandiaode	1.not interesting; showing very little imagination<br>2.having no excitement, interest, or new and different events
91	轻松的	Qingsongde	1.entertaining and easily understood, but not serious and not intended to make you think<br>2.used for describing things that are not serious and that are easy to understand and enjoy
92	令人安心的	Lingrenanxinde	1.If you are comfortable with a situation, you are not worried about it<br>2.comfortable or calm; free from worry, pain, etc

93	协调的	Xietiaode	able to exist, live together, or work successfully with something or someone else
94	解压的	Jieyade	X
95	舒适的	Shushide	1.used to describe a pleasant or comfortable place or thing<br>2.A friendly place is pleasant and makes you feel happy and comfortable<br>3.A relaxed situation or place is comfortable
96	优雅的	Youyade	ight and delicate, especially in an unnatural way
97	惬意的	Qieyide	1.comfortable and pleasant, especially (of a building) because of being small and warm<br>2.used to describe a pleasant or comfortable place or thing
98	美好的	Meihaode	1.pleasant, enjoyable, or satisfactory<br>2.a situation in which everything is pleasant and easy<br>3.extremely good, pleasant, or enjoyable
99	令人平复的	Lingrenpingfude	of a feeling) made less strong or to bear
100	治愈的	Zhiyude	helping to make someone well again, especially after a cut or other injury
101	宜人的	Yirende	1.enjoyable, attractive, friendly, or easy to like<br>2.friendly and pleasant
102	平静的	Pingjingde	1.having little activity or excitement and few people<br>2.calm and peaceful and without noise, violence, worry, etc<br>3.peaceful and calm; worried by nothing
103	舒心的	Shuxinde	X
104	闲适的	Xianshide	X
105	温柔的	Wenroude	calm, kind, or soft
106	悠闲的	Youxiande	1.relaxed and not easily upset or worried<br>2.slow and relaxed
107	和谐的	Hexiede	1.in a style that is suitable, goes well with other things that are in the same place, and considers the feelings and needs of people who use that place
108	愉悦的	Yuyuede	1.the quality of being enjoyable and pleasant to do<br>2.the quality of being very attractive and pleasing to hear, look at, read, feel, etc

Table S10 108(108-7) Soundscape attributes the 16 clusters.

Clustering	Soundscape Attributes Members
C1	令人紧张的、有压迫感的、压抑的、奇怪的、令人沮丧的
C2	有画面感的、有生活气息的、身临其境的
C3	有趣的、令人开心的、令人愉快的、令人欢乐的、吸引人的、放松的、平和的、有吸引力的、轻松的、令人安心的、协调的、舒适的、惬意的、美好的、治愈的、宜人的、舒心的、闲适的、悠闲的、愉悦的
C4	内容丰富的、热闹的、生气勃勃的、有活力的、有故事性的、引人遐想的
C5	令人困倦的、沉闷的、无变化的、无体验感的、单调的
C6	令人欣喜的、有韵律的、舒缓的、悦耳的、动听的
C7	温和的、宁静的、柔和的、恬静的、解压的、令人平复的、平静的、温柔的
C8	生动的、有生机的、富有情感的、幸福的、亲切的、有感染力的、和谐的
C9	嘈杂的、喧嚣的、喧闹的、吵闹的
C10	无趣的、枯燥的、平淡无奇的、无聊的、乏味的
C11	空灵的、优美的、优雅的
C12	令人振奋的、令人兴奋的、令人激动的
C13	有氛围感的、引发联想的、引起回忆的、熟悉的
C14	混乱的、有干扰性的
C15	寂静的、冷清的、凄凉的、沉寂的、静谧的、安静的
C16	令人不舒服的、令人厌烦的、令人讨厌的、烦人的、恼人的、难听的、令人烦恼的、令人不适的、令人烦躁的、令人焦躁的、令人不快的、令人反感的、令人抓狂的、糟糕的、令人抵触的

Clustering	Soundscape Attributes Members
C1	lingrenjinzhangde; youyapogande; yayide; qiguaide; lingrenjusangde
C2	youhuamiangande; youshenghuoqixide; shenlinqijingde
C3	youqude; lingrenkaixinde; lingrenyukuaide; lingrenkuailede; xiyinrende; fangsongde; pinghede; youxiyinlide; qingsongde; lingrenanxinde; xietiaode; shushide; qieyide; meihaode; zhiyude; yirende; shuxinde; xianshide; youxiande; yuyuede
C4	neirongfengfude; renaode; shengjibobode; youhuolide; yougushixingde; yinrenxiaxiangde
C5	lingrenkunjuande; chenmende; wubianhuade; wutiyangande; dandiaode
C6	lingrenxinxide; youyunlvde; shuhuande; yueerde; dongtingde
C7	wenhede; ningjingde; rouhede; tianjingde; jieyade; lingrenpingfude; pingjingde; wenroude
C8	shengdongde; youshengjide; fuyouqinggande; xingfude; qinqiede; youganranlide; hexiede
C9	caozade; xuanxiaode; xuannaode; chaonaode
C10	wuliaode; kuzaode; pingdanwuqide; wuliaode; faweide
C11	konglingde; youmeide; youyade
C12	lingrenzhenfende; lingrenxinfende; lingrenjidongde
C13	youfenweigande; yinqilianxiangde; yinqihuiyide; shuxide
C14	hunluande; youganraoxingde
C15	jijingde; lengqingde; qiliangde; chenjide; jingmide; anjingde
C16	lingrenbushufude; lingrenyanfande; lingrentaoyande; fanrende; naorende; nantingde; lingrenfannaode; lingrenbushide; lingrenfanzaode; lingrenjiaozaode; lingrenbukuaide; lingrenfangande; lingrenzhuakuangde; zaogaode; lingrendichude
